# The Prevalence and Impact of Heavy Menstrual Bleeding (Menorrhagia) in Elite and Non-Elite Athletes

**DOI:** 10.1371/journal.pone.0149881

**Published:** 2016-02-22

**Authors:** Georgie Bruinvels, Richard Burden, Nicola Brown, Toby Richards, Charles Pedlar

**Affiliations:** 1 Division of Surgery and Interventional Science, University College London, London, United Kingdom; 2 School of Sport, Health and Applied Science, St Mary’s University, Twickenham, United Kingdom; 3 Orreco Ltd, Institute of Technology, Sligo, Ireland; 4 English Institute of Sport, Loughborough, United Kingdom; Oklahoma State University, UNITED STATES

## Abstract

To identify the prevalence and impact of heavy menstrual bleeding (HMB) in exercising females where anemia may have a significant effect on training and performance a ‘Female Health Questionnaire’ was designed incorporating a validated diagnostic HMB series, demographics, exercise ability data, training status, anemia, iron supplementation and whether the menstrual cycle had affected training and performance. The survey was conducted in two stages; initially online, advertised via social media, and then repeated via face-to-face interviews with runners registered for the 2015 London Marathon. 789 participants responded to the online survey, and 1073 completed the survey at the marathon. HMB was reported by half of those online (54%), and by more than a third of the marathon runners (36%). Surprisingly, HMB was also prevalent amongst elite athletes (37%). Overall, 32% of exercising females reported a history of anemia, and 50% had previously supplemented with iron. Only a minority (22%) had sought medical advice. HMB is highly prevalent in exercising females, associated with self-reported anemia, increased use of iron supplementation and a perceived negative impact on performance. Further research is needed to investigate the impact of HMB, iron deficiency and anemia in exercising females.

## Introduction

Heavy menstrual bleeding (HMB) is common, affecting a quarter of the female population.[1] HMB can negatively impact on physical, emotional and social quality of life and reduce work capacity.[2,3]

Diagnosing HMB can be subjective and definitions include; blood loss of more than 80ml per menstrual cycle or “excessive menstrual blood loss which interferes with a woman’s physical, social, emotional and/or material quality of life”.[3,4] In a recent Europe-wide study a diagnosis of HMB was given if two or more of the following criteria were met; 1. passing of large blood clots, 2. need for double sanitary protection (both towels and tampons), 3. need for frequent changes of tampons and towels (meaning changes every 2 hours or less, or 12 sanitary items per period) and 4. flooding through to clothes or bedding.[1]

The greater blood loss in HMB increases susceptibility to iron deficiency, which if left untreated may progress to iron deficiency anemia (IDA). Iron is an essential micronutrient required for numerous biological functions, including oxygen transport, cellular and mitochondrial respiration, electron transfer reactions, gene regulation, cell growth and differentiation.[5] Compromised iron stores cause adaptive changes, eventually resulting in limitations to the production of hemoglobin and a state of IDA. Menstruation is the most common single cause of IDA in females of a childbearing age,[6] with HMB specifically identified as the principal cause of iron deficiency and IDA in clinical practice in this population.[7] In a recent study of women with HMB, 63% of respondents reported being deficient in iron at some point.[1] However, despite the high prevalence of HMB, awareness is poor, with only a small minority (6%) of women seeking medical help annually.[8]

The impact of compromised iron stores on oxidative metabolism in endurance athletes can be significant, potentially reducing total hemoglobin mass, oxygen carrying capacity and performance.[9,10] Furthermore, those who exercise are at higher risk of iron deficiency as a result of increased iron loss through hematuria (blood in urine), gastrointestinal bleeding, sweating and hemolysis (particularly exacerbated in impact sports involving foot strike).[11–14] Research into the impact of iron deficiency without anemia is inconclusive, with an identified need for further research.[15]

While HMB has been shown to affect more than a quarter of women in the general population, the prevalence of HMB and the impact upon training and performance in exercising females has been unknown. We have recently published this headline data in a brief letter,[16] and in this paper we aim to 1. provide the full methods and results from this study, identifying the prevalence of HMB in exercising females; 2. determine any differential effect on exercisers of varying abilities; and 3. outline the perceived reported impact of HMB on training and performance which we were able to do through this research.

## Materials and Methods

This research has been approved by the St Mary's University Ethics Committee. A 12-item ‘Female Health Questionnaire’ including free-text and yes-no polar questions was developed and designed to take 2–3 minutes to complete. The four-symptom definition of HMB [1] was used to identify HMB sufferers and information was collected on age, ‘personal best’ sports performance times, current training volume, previous history of anemia and iron supplementation (including as part of a multivitamin), the menstrual cycle and difficulties caused by it, and oral contraceptive pill (OCP) usage (S1 Appendix). The participants were informed that by indicating that they agree to the terms and completing the survey they have provided written informed consent for their information to be used in this study. The inclusion criteria were: female, aged ≥18 years, pre-menopausal and regularly exercising (≥90 minutes/week).

### Stage 1—online questionnaire

The questionnaire was administered online and advertised through social media including Twitter, Facebook, online blogs and forums, university newsletters, websites and by word of mouth between 22 January 2015 and 19 May 2015. A link was provided to the internet-based survey in addition to some brief information about the research.

### Stage 2—marathon exhibition questionnaire

Females registering for the 2015 London Marathon at the pre-event exhibition were surveyed using the same questionnaire. No bias was applied when selecting females to question and to avoid a response bias a scripted standardized introduction was made providing no specific information about the context of the survey. To ensure maximum response yield, surveys were completed at the time of asking. The questions and format of the paper copies used at the Exhibition were identical to the online survey to maintain equivalency and reliability of this mixed mode strategy.[17]

### Data Analysis

Data were analyzed descriptively to summarize the prevalence of HMB, known anemia, iron supplementation, the seeking of medical help and impact of HMB on training and performance in both stages 1 and 2. The statistical analysis was completed using a predictive analytic software statistics computer package (IBM SPSS Statistics for Macintosh, Version 21.0, Armonk, NY: IBM Corp.). Statistical significance was set at P<0.05. Chi-squared tests were used to determine whether there was an association between HMB and presence of anemia and HMB and self-reported impacts on training and performance. Mann-Whitney U and Kruskal Wallis H tests were used to determine whether age and average weekly training volume were related to HMB. A Kruskal-Wallis H test with *post hoc* analysis and correction was used to determine whether 5km personal best time was linked to the number of HMB symptoms experienced, and Mann-Whitney U tests and Chi-squared tests were used to determine whether participant performance level (based on 5km personal best) was related to HMB incidence.

After combining both groups, a sub-analysis was conducted to separate out elite athletes using the following criteria: 5km ≤18 minutes, 10km ≤36 minutes, half marathon ≤80 minutes, 2km row ≤7 minutes:45 seconds (elite running criteria defined using the 2015 ‘Great Run’ series definitions of ‘elite’, rowing criteria defined by English Institute of Sport physiologist). Participants were split into the following groups based on typical total minutes exercised per week <90, 90–180, 180–360, 360–540, 540–720, and >720 minutes.

## Results

### Stage 1

A total of 789 surveys were completed online. More than half (54.1%) of the participants had experienced HMB at some point (Table 1).[16] 55.4% stated that their menstrual cycle impacts upon their training and performance (Table 1), with those meeting the HMB criteria (n = 427) being more likely to state this (69.3% vs. 39.0%; *χ2 = 867*.*593*, *p<0*.*01)* (Table 2).[16] Those with a history of HMB were found to be older (31 years ±9.32 vs. 29 years ±7.49; *H(2) = 10*.*392*, *p<0*.*01)*.

**Table 1:** Self reported prevalence of heavy menstrual bleeding (HMB), the effects on training and performance, seeking of help, history of anemia and iron supplementation. [16]

	Stage 1 (n = 789)	Stage 2 (n = 1073)	Elite athletes (n = 90)
HMB	427 (54.1%)	381 (35.5%)	33 (36.7%)
Affects training and performance	437 (55.4%)	340 (31.7%)	46 (51.1%)
Sought help	190 (24.1%)	226 (21.1%)	21 (23.3%)
History of anemia	303 (38.4%)	300 (28.0%)	47 (52.2%)
History of iron supplementation	451 (57.2%)	486 (45.3%)	71 (78.9%)

**Table 2:** Self reported prevalence of heavy menstrual bleeding (HMB), its effects on training and performance, seeking of help, history of anemia and iron supplementation in those who have met the HMB criteria.

	Stage 1 (n = 427)	Stage 2 (n = 381)	Elite athletes (n = 33)
Affects training and performance	296 (69.3%)*	184 (48.3%)*	22 (66.7%)***
Sought help	159 (37.2%)*	170 (44.6%)*	14 (42.4%)**
History of anemia	184 (43.1%)*	145 (38.1%)*	19 (57.6%)
History of iron supplementation	262 (61.4%)***	210 (55.1%)*	27 (81.8%)

Significant differences between the values here from those meeting the HMB criteria and those who haven’t are shown as follows:

*p < 0.001,

**p < 0.01,

***p < 0.05.

Of the 427 participants who met the HMB criteria, 37.2% had sought medical help for heavy periods (Table 2).

### Stage 2

1091 face-to-face surveys were collected and inputted into the Bristol Online Survey platform manually by the lead investigator and an assistant. Those with missing data or those completed by females who did not meet the inclusion criteria were excluded, resulting in a final sample size of 1073 for further analysis. Eight individuals declined to complete the survey once they had read the study information, and 61 declined answering the survey prior to being informed about the content typically citing a lack of time. Therefore, in stage 2, the survey was fully completed by 94% of randomly approached female marathon runners.

The prevalence of HMB in females undertaking the 2015 London Marathon was 35.5% (Table 1).[16] Overall nearly one third (31.7%) said that their menstrual cycle impacts upon their training and performance (Table 1).[16] This was more than twice as likely to be a problem in those with HMB (48.3% vs. 22.5%; *χ2 = 1151*.*481*, *p<0*.*01*; Table 2). Those who have experienced HMB were older than those who have not (35 years ±7.95 vs. 32 years ±7.83; *H(2) = 18*.*936*, *p<0*.*01)*. Of the 381 participants who met the criteria for HMB, 44.6% had sought medical help (Table 2).

Across both groups, known anemia was reported by 603 (32.4%) participants, while 1049 (56.3%) specified that they were unsure whether they have had anemia.[16] Reported anemia was more common in those with HMB (40.7% vs. 26.0%; *χ2 = 70*.*765*, *p<0*.*01*).[16] Use of iron supplementation was also more common in those reporting HMB (58.4% vs. 50.3%; *χ2 = 39*.*199*, *p<0*.*01)*.[16] Less than a quarter of all surveyed reported having sought help for heavy periods (22.3%), with this increasing in those who met the HMB criteria (40.7%).[16]

When a sub-analysis was conducted and elite athletes were separated out from both groups, 36.7% met the HMB criteria, with 51.1% indicating that their menstrual cycle has impacted upon their training and performance, with these being significantly related (*χ2 = 5*.*046*, *p<0*.*05*). A history of anemia was reported by 52.2%, with 78.9% having supplemented with iron (Table 1).

**Fig 1:**
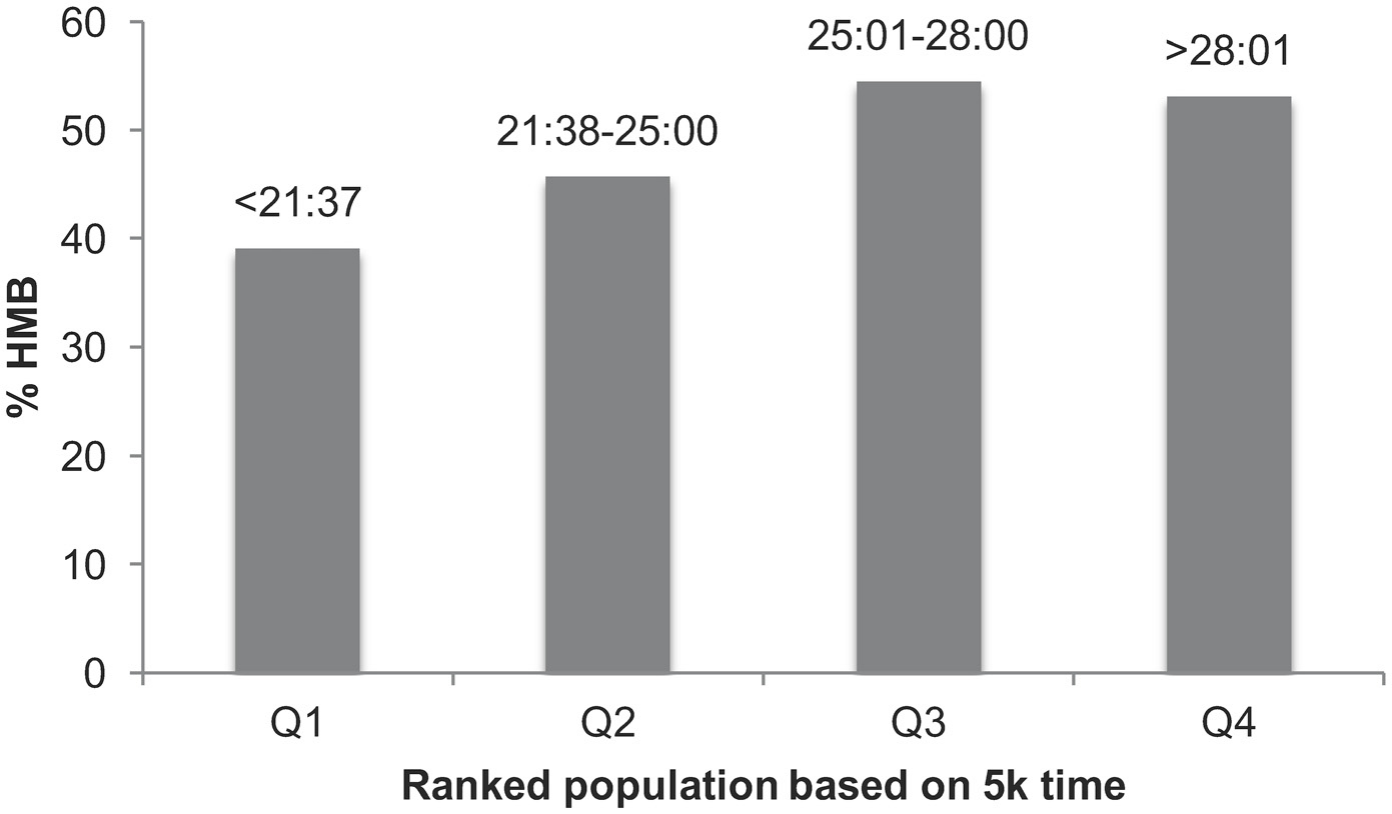
Prevalence of reported heavy menstrual bleeding (HMB) across participant performance level. 5km personal best times (minutes:seconds) are divided into quartiles, Q1 representing those with the fastest times, Q4 the slowest. A significant difference was found between groups *(p < 0*.*01)*.

When participants who specified a 5km personal best time (n = 1166) were divided into groups based on the number of HMB symptoms they have experienced, a significant difference was found between groups (*H(4) = 11*.*464*, *p<0*.*05*), despite distributions looking similar. However, a *post hoc* analysis using pairwise statistics revealed no statistically significant pairwise comparisons. When simply comparing those with and without HMB, median 5km times were significantly different (25 minutes:0 seconds vs. 24 minutes:24 seconds; *z = 3*.*099*, *p<0*.*05*). When 5km personal best times were divided into quartiles (Q1 being the fastest athletes), a significant difference was seen in HMB prevalence between groups *(χ2 = 14*.*890*, *p<0*.*01)*, the faster runners in Q1 being less likely to have HMB (39.1%) than the slower runners in Q4 (53.1%) (Fig 1).

No statistically significant association was found between average weekly exercise volume and HMB presence (*z = -0*.*811*, *p>0*.*05*). Those exercising for >720 minutes each week appeared as likely to suffer from HMB as those exercising for <90 minutes each week (*χ*^*2*^ = *6*.*765*, *p>0*.*05)*.

## Discussion

This is the first study to identify that HMB is a common problem amongst exercising females. Stage 1 of this research was used to ascertain whether HMB was prevalent amongst exercising females. It is acknowledged that this online survey was likely to be biased because females with menstrual cycle issues were more likely to complete the questionnaire, however with 54.1% meeting the HMB criteria this demonstrated that this is a significant problem within this populace. To obtain unbiased prevalence data a large study (stage 2) incorporating a number of controls to prevent bias was conducted at the 2015 London Marathon Exhibition. This found that 35.5% marathon runners met the HMB criteria therefore confirming the outcome in stage 1 that this is a common problem amongst exercising females. HMB has previously been shown to affect more than a quarter of the general female population,[1] but this is the first study to investigate prevalence amongst exercising females.

Only 43.1% and 38.1% of those females from stages 1 and 2 with HMB had sought medical help. This highlights the need for increased HMB awareness. However, previous research has demonstrated that almost half of females meeting the criteria for HMB who sought help did not have HMB confirmed,[1] and the results of an audit conducted by the Royal College of Obstetricians showed that once diagnosed one third were not given treatment in primary care.[18] This suggests that from both a diagnostic and treatment perspective an increased awareness of HMB prevalence and treatment options is required. However, a review of treatment methods has shown there to be considerable variation in the treatment procedures and medications used, highlighting the need for further research.[19]

Due to the increased blood loss, those with HMB are more likely to suffer from iron deficiency and anemia, and this is consistent with our finding that those meeting the HMB criteria were more likely to report previous diagnosis of anemia than those who do not (40.7% compared to 26.0%). This may be higher as more than half of all respondents said that they were unaware whether or not they have been anemic. In the general population IDA has been shown to affect two thirds of women with HMB.[3] Regardless of menstruation, those participating in endurance exercise are susceptible to iron deficiency due to increased iron losses as a result of foot strike hemolysis, sweating, and gastrointestinal bleeding.[11–14] Dietary intake of iron has also been found to be suboptimal in those who exercise, and particularly in females.[20] This iron deficiency and IDA risk is further exacerbated in those with HMB. Many elite athletes routinely supplement with iron—as shown here with 78.9% reporting supplementation. Coaches often encourage supplementation without knowledge of iron status due to the unfounded but common belief that iron deficiency is rife and supplementation may benefit performance. Less than half of those with HMB have sought medical help, therefore it is necessary to raise awareness as clinical iron deficiency and IDA can result in fatigue, weakness, impaired cognition and psychological morbidity, negatively impacting upon quality of life.[21]

Similar to the findings from Marret et al,[22] this study demonstrated that HMB was marginally more likely in those who were older. Unsurprisingly those with HMB were more likely to report that their menstrual cycle impacts upon their training and performance.

The sub-analysis from the elite athlete sample showed that more than one third met the HMB criteria. This is somewhat surprising because it is well documented that elite female athletes, particularly endurance athletes are susceptible to amenorrhea and oligomenorrhea often as a result of a relative energy deficiency associated with a high training volume.[23,24] This suggests that elite athletes may also be susceptible to other menstrual disturbances. Furthermore, it could be hypothesized that increases in training volume would equate to increased risk of amenorrhea or oligomenorrhea, potentially decreasing HMB incidence, but this was not the case here with no identified relationship between total number of minutes exercised per week and HMB presence. However, those with faster 5km personal best times were less likely to report HMB, with the median 5km time in the HMB group being slower. The slower times seen in Q3 and Q4 where HMB prevalence is higher when compared to Q1 could be caused by an increased incidence of IDA, which is impacting upon performance, alternatively increased rates of amenorrhea in Q1 could reflect the lower HMB incidence seen here. However these differences were only marginal, and further research is required before forming a definitive conclusion. Historically, much research has focused on the female athlete triad and the new term ‘Relative Energy Deficiency in Sport’–RED-S, particularly in elite athletes.[25,26] These syndromes are characterized by amenorrhea or oligomenorrhea, however this study suggests that other menstrual cycle issues are also commonplace, highlighting the need for further more general research across other menstrual cycle irregularities in exercising women.

There are a number of limitations of this study. Firstly, the self-reported nature of this questionnaire could have resulted in inaccurate data, however the HMB diagnostic criteria does not lean itself to comparison bias. Secondly, stage 2 data was only collected in marathon runners, which may not be representative of other running events and sports. It has been shown that exercise increases susceptibility to iron deficiency,[27] however blood parameters including markers of iron status have not been measured therefore we are making an assumption. Additionally, the presence of illnesses (i.e. endometriosis) or the use of medication was not recorded, these could increase the likelihood of participants meeting the HMB criteria. To obtain standardized performance comparisons a means for knowing finishing time in the marathon would strengthen ability to determine any relationships between participant performance level and HMB presence. Relationship between anthropometrical parameters and and HMB presence could also be explored. Further studies are required to address these limitations.

## Conclusions

This study has demonstrated that HMB is common in the exercising population. HMB was associated with anemia, iron supplementation and slower performance times. Further research is however needed to explore the impact of HMB and iron deficiency on performance. The lack of medical help sought by the participants in this study suggests that either females don’t feel or realize this is a problem, or have learnt to cope with it, highlighting that more research and awareness is needed. HMB is also surprisingly common amongst elite athletes, ostensibly impacting upon their training and performance, and potentially causing iron deficiency, although further research is needed to confirm this association.

## Supporting Information

S1 Appendix ‘Female Health Questionnaire’. The ‘Female Health Questionnaire’ that was completed either online or at the 2015 London Marathon Exhibition by all surveyed (n = 1862).
(DOCX)

S2 Appendix ‘Letter to the Editor’. Letter to the Editor: The prevalence and impact of heavy menstrual bleeding amongst athletes and mass start runners of the 2015 London Marathon.
(DOCX)
