# Supplementary material for: The Prevalence and Impact of Heavy Menstrual Bleeding (Menorrhagia) in Elite and Non-Elite Athletes
Source: PLoS One. 2016 Feb 22;11(2):e0149881. doi: 10.1371/journal.pone.0149881 (PMC4763330; doi:10.1371/journal.pone.0149881)
Supplement: S1 Appendix — The ‘Female Health Questionnaire’ that was completed either online or at the 2015 London Marathon Exhibition by all surveyed (n = 1862). (DOCX) [file pone.0149881.s001.docx]

**S1 Appendix – ‘Female Health Questionnaire’**

**
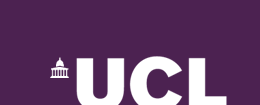
**

**
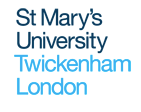
**

**Female Health Questionnaire**

The research group at St Mary’s University, London in collaboration with University College London are investigating iron status in endurance athletes. One of the key factors considered to impact upon iron status in female athletes is blood loss during the menstrual cycle. This is further exacerbated in those who experience larger blood losses (heavy menstrual bleeders). Currently little research has been conducted in this area, therefore we are conducting a survey-based study firstly to look at the prevalence of menstrual issues amongst female athletes and secondly to investigate how the menstrual cycle affects training and performance. Could all women who read this please answer the survey – not just those who feel they might have an issue with their menstrual cycle.

In order to participate in this study you must be:

- Female
- 18 years or older
- Pre-menopausal

By completing this survey you are giving consent for the information you provide to be included in this study. Your participation is voluntary and is specific to this study, and shall not be taken to imply consent to participate in any subsequent experiment or deviation from that detailed. All information will remain confidential as to your identity, and you may withdraw from the study at anytime without reason.

If there is anything you do not understand or wish to ask questions about, please feel free to ask.

Thank you for participating

YES NO

Do you agree to these terms?

1. Age

1. How much time do you spend exercising each week?

|  | Time (minutes) | |
| --- | --- | --- |
| Running |  |  |
| Cycling |  |  |
| Swimming |  |  |
| Other |  |  |

1. Please specify your personal best times for all/any of the below in the last year:

|  | Time (minutes) |
| --- | --- |
| 5km run (inc Parkrun) |  |
| 10km run |  |
| Half marathon |  |
| 10M TT cycle |  |
| 25M TT cycle |  |
| 2km row |  |

1. Approximately how many periods have you had in the last year?

1. Have you ever sought advice/help for heavy periods?

| YES |  |  | NO |  |
| --- | --- | --- | --- | --- |

1. To your knowledge, have you ever had anaemia?

| YES |  |  | NO |  |  | DON’T KNOW |  |
| --- | --- | --- | --- | --- | --- | --- | --- |

1. Have you ever supplemented with iron?

| YES |  |  | NO |  |  | DON’T KNOW |  |
| --- | --- | --- | --- | --- | --- | --- | --- |

1. Have you ever experienced any of the following? (*tick all that apply)*

| Flooding through to clothes or bedding |  |
| --- | --- |
| Need of frequent changes of sanitary towels or tampons |  |
| *(meaning changes every 2 hours or less, or 12 sanitary items per period)* |  |
| Need of double sanitary protection *(tampons and towels)* |  |
| Pass large blood clots |  |

1. Do you currently use the oral contraceptive pill?

| YES |  |  | NO |  |
| --- | --- | --- | --- | --- |

1. Do you feel that your menstrual cycle disrupts your training/performance?

| YES |  |  | NO |  |
| --- | --- | --- | --- | --- |

1. Date of birth: *(dd/mm/yyy)*
2. We will be conducting more research in this area. If you are happy to be contacted further please provide your email address:

Supplementary Material S1 – the ‘Female Health Questionnaire’ that was completed either online or at the 2015 London Marathon Exhibition by all females surveyed (n=1862).
