# Supplementary material for: The Prevalence and Impact of Heavy Menstrual Bleeding (Menorrhagia) in Elite and Non-Elite Athletes
Source: PLoS One. 2016 Feb 22;11(2):e0149881. doi: 10.1371/journal.pone.0149881 (PMC4763330; doi:10.1371/journal.pone.0149881)
Supplement: S2 Appendix — Letter to the Editor: The prevalence and impact of heavy menstrual bleeding amongst athletes and mass start runners of the 2015 London Marathon. (DOCX) [file pone.0149881.s002.docx]

**S2 Appendix – ‘Letter to the Editor’**

**Letter to the Editor: The prevalence and impact of heavy menstrual bleeding** **amongst athletes and mass start runners of the 2015 London Marathon**

Georgie Bruinvels^1,2,3^, Richard Burden^2,4^, Nicola Brown^2^, Toby Richards^1^, Charles Pedlar^2,3^

^1^*University College London*, ^2^*St Mary’s University, Twickenham* ^3^Orreco Ltd. ^4^English Institute of Sport, UK

Corresponding author: [georgie.bruinvels.14@ucl.ac.uk](mailto:georgie.bruinvels.14@ucl.ac.uk) @gbruinvels

Postal Address: School of Sport, Health and Applied Science, St Mary’s University, Waldegrave Road, Twickenham, London TW1 4SX

Telephone number: +44(0)7917 652179

Dr Richard Burden, School of Sport, Health and Applied Science, St Mary’s University, London, UK

Dr Nicola Brown, School of Sport, Health and Applied Science, St Mary’s University, London, UK

Professor Toby Richards, Division of Surgery and Interventional Science, University College London, London, UK

Dr Charles Pedlar, School of Sport, Health and Applied Science, St Mary’s University, London, UK

**Key words:** Heavy Menstrual Bleeding Iron Deficiency

**Word Count:** 505 words

**Acknowledgements**: The authors thank Dr Courtney Kipps and the London Marathon Medical Committee for their cooperation in facilitating access to the London Marathon Exhibition.

**Contributors**: GB participated in protocol design, data collection, data analyses and manuscript preparation. CP, TR, RB and NB participated in the protocol design, data analysis and manuscript preparation.

**Competing interests**: None

**Ethical Approval**: St Mary’s University, Twickenham Ethics Committee.

**Provenance and Peer Review**: Not commissioned; externally peer reviewed

The single most common cause of iron deficient anaemia in the developed world in premenopausal females is the menstrual cycle.[1] It is well recognised and reported that amenorrhea and oligomenorrhea are common in elite athletes typically as a result of relative energy deficiency,[2] however, little is known about the prevalence of other menstrual abnormalities. Heavy menstrual bleeding (HMB or *menorrhagia*) affects a quarter of the general population,[3] yet no data exist for athletes or exercising women. It is possible that HMB might impact significantly upon women’s participation in sport. HMB can lead to fatigue, anxiety, reduced mood and energy levels with a negative impact on quality of life and productivity.[4] Furthermore, iron turnover in exercising females is likely to be increased further due to factors such as haemolysis putting them at a high risk of iron deficiency anaemia.[5] Iron is an essential micronutrient required for numerous biological functions, and deficiency can result in adaptive changes limiting haemoglobin production and a state of iron deficiency anaemia. We sought to identify the prevalence and impact of HMB in exercising females where anaemia may have a significant effect on training and performance.

We recently conducted a ‘Female Health Questionnaire’, which incorporated a validated diagnostic HMB series, demographics, athlete ability data, training status, known anaemia, iron supplementation and questions concerning the effect of the menstrual cycle on training and performance. The survey was initially conducted online (n=789 women), advertised via social media. Subsequently, to obtain non-biased data, the same survey was then conducted via face-to-face interviews with runners during registration for the 2015 London Marathon Exhibition (n=1073 women). Among the group a total of 90 participants were classified as ‘elite’. The key findings from this survey were that HMB was common in both groups; reported by over half of those online (54%), and by more than a third of the marathon runners (36%). 55% (online) and 32% (marathon runners) stated that their menstrual cycle impacted upon training and performance, this being more common in those with HMB (*χ2=183.4, p<0.01*). Surprisingly, HMB was also prevalent amongst elite athletes (37%). Overall, 32% of all participants reported a history of anaemia, with this also being more common in those who have experienced HMB (41% vs. 26%; *χ2=70.765, p<0.01)*, while 50% had previously supplemented with iron. Only a minority (22%) had sought medical advice. No significant association was found between average weekly exercise volume and HMB presence.

In summary, we found HMB to be highly prevalent in female athletes, associated with anaemia, an increased use of iron supplementation and reported negative impacts on performance. Somewhat unexpectedly, our results suggest that HMB is more common in the exercising population than in the general population. Although there are a number of limitations to this questionnaire based study, we highlight that HMB may be under-recognised. Further research is needed to describe this issue and to understand its implications. Interventions to support female athletes such as iron therapy,[6] and an increased awareness of HMB amongst Sports Medicine professionals could have far reaching benefits for the female athlete.

**References**

1 Annibale B, Lahner E, Chistolini A, et al. Endoscopic evaluation of the upper gastrointestinal tract is worthwhile in premenopausal women with iron-deficiency anaemia irrespective of menstrual flow. Scand J Gastroenterol 2003;38:239-45

2 Mountjoy M, Sundgot-Borgen J, Burke L et al. The IOC consensus statement: beyond the Female Athlete Triad –Relative Energy Deficiency in Sport (RED-S). Br J Sports Med 2014;48:491-7.

3 Fraser IS, Mansour D, Breymann C, et al. Prevalence of heavy menstrual bleeding and experiences of affected women in a European patient survey. Int J Gynaecol Obstet 2015;128:196-200.

4 Karlsson TS, Marions LB, Edlund MG. Heavy menstrual bleeding significantly affects quality of life. Acta Obstet Gynecol Scand 2014;93:52–7.

5 Telford RD, Sly GJ, Hahn AG, et al. Footstrike is the major cause of hemolysis during running. J Appl Physiol 2003;94:38-42.

6 Burden RJ, Morton K, Richards T, et al. Is iron treatment beneficial in iron-deficienct but non-anaemic (IDNA) endurance athletes: A meta-analysis. Br J Sports Med 2014; pii: bjsports-2014-093624. doi: 10.1136/bjsports-2014-093624. [Epub ahead of print] Review
